# Recent advances in exploring and exploiting soybean functional peptides—a review

**DOI:** 10.3389/fnut.2023.1185047

**Published:** 2023-06-15

**Authors:** Yongsheng Zhu, Gang Chen, Jingjing Diao, Changyuan Wang

**Affiliations:** ^1^Hangzhou Joyoung Soymilk & Food Co., Ltd., Hangzhou, China; ^2^National Coarse Cereals Engineering Research Center, Heilongjiang Bayi Agricultural University, Daqing, China; ^3^College of Food Science, Heilongjiang Bayi Agricultural University, Daqing, China

**Keywords:** soybean, functional peptides, plant food, health, fermentation

## Abstract

Soybeans are rich in proteins and phytochemicals such as isoflavones and phenolic compounds. It is an excellent source of peptides with numerous biological functions, including anti-inflammatory, anticancer, and antidiabetic activities. Soy bioactive peptides are small building blocks of proteins that are released after fermentation or gastrointestinal digestion as well as by food processing through enzymatic hydrolysis, often in combination with novel food processing techniques (i.e., microwave, ultrasound, and high-pressure homogenization), which are associated with numerous health benefits. Various studies have reported the potential health benefits of soybean-derived functional peptides, which have made them a great substitute for many chemical-based functional elements in foods and pharmaceutical products for a healthy lifestyle. This review provides unprecedented and up-to-date insights into the role of soybean peptides in various diseases and metabolic disorders, ranging from diabetes and hypertension to neurodegenerative disorders and viral infections with mechanisms were discussed. In addition, we discuss all the known techniques, including conventional and emerging approaches, for the prediction of active soybean peptides. Finally, real-life applications of soybean peptides as functional entities in food and pharmaceutical products are discussed.

## Introduction

Soybean (*Glycine max*) is a legume of the family Fabaceae (Leguminosae), which originated ~5,000 years ago in East Asia. Currently, the USA, Brazil, and Argentina account for ~34, 30, and 17% of the global soybean production, respectively. China, Japan, Mexico, and many European countries are the main soybean-importing countries, and their export products are oil, meal, and seed, which account for 80% of their known export value (1). Standard soybean seeds with 13% of moisture comprise 19% oil and 68% meal fractions (2, 3).

Soybean is a potent source of vegetable proteins and phytochemicals, such as isoflavones and phenolic compounds, because of its amino acid composition, high availability, and low cost, making it the second largest source of vegetable oil globally (4, 5). It consists of protein (30–35%), lipids (20%), dietary fiber (9%), and moisture (8.5%) based on the dry weight of soybean seeds (6). Various environmental conditions and genetic modifications affect the soybean protein composition which has an influence on soybean health-promoting properties for the production of functional soybean-based products (7). Albumins and globulins are the two main proteins in soybean, and the latter is considered the main protein component of soybeans. The two major storage proteins of soybean are β-conglycinin (βCG, 7S) and glycinin (11S), which account for 80–90% of the total protein in soybean (8–10). Soybean dietary proteins have been shown to have health benefits owing to their therapeutic nature as a result of the presence of functional peptides (11, 12). Functional motifs of a protein are termed peptides. Soybean-derived peptides have gained considerable interest because of their potential health benefits associated with metabolism, brain function, and cognitive ability, as well as their immunomodulatory, antioxidative, antithrombotic, anti-inflammatory, antihypertensive, antidiabetic, antiviral, and mineral-binding roles (13–17).

Soybean-derived peptides are also known to attenuate the severity of metabolic and age-related chronic disorders, such as cancer, hypocholesterolemia, obesity, and alcohol-induced liver injury (18–20). Several peptides derived from soybeans have been reported to exert multiple benefits and are termed multifunctional soybean peptides. Lunasin (5.5 kDa molecular weight), comprising 43 amino acids, is a multifunctional soybean peptide (21). This peptide has been reported to ameliorate several chronic diseases and metabolic conditions including cancer, hypertension, oxidative stress, and inflammation (22, 23). It is often utilized as a dietary supplement because of its high availability and heat stability.

Apart from the plethora of functional peptides in soybeans, isoflavones are also present as glycosides (24). They can lower the risk of oxidative damage to DNA and low-density lipoprotein (LDL) by free radical stress and increase the antioxidant function of enzymes that play a role in the defense system of the human body. They can bind to reactive oxidative species, improve glutathione production, and vitamins E and C, which also work effectively as antioxidants (25, 26). Moreover, these isoflavones can decrease cancer promoters that produce oxidative stress, such as xanthine and tetradecanoylphorbol-13-acetate (TPA) (27). Isoflavones are reported to act as potential antidepressants (28). Genistein is a widely existing and well-known isoflavone, which is associated with a decreased risk of various diseases that are linked to humans, such as tumors, by the enzymatic intervention (29). Genistein has been shown to be helpful in the regulation of gene transcription through DNA methylation and histone modification (30).

Soybeans are known to be a complete protein because they contain all the five key amino acids required for proper nutrition. The traditional methods of acquiring these peptides include the following techniques: (i) hydrolytic activities of enzymes (e.g., trypsin, pepsin, and papain) (31), (ii) microbial fermentation by *Lactobacillus* and yeast (32, 33), (iii) combined enzymatic and microbial treatment (34), and (iv) food processing by high pressure and enzymatic hydrolysis (35). Enzymatic hydrolysis is the most convenient and common method because of its high stability and production of many other useful molecules during fermentation, such as surfactants, bacteriocins, polysaccharides, amino acids, vitamins, and many other biomolecules (36). Lactic acid fermentation has proven to be the best method for bioactive peptide production and protein hydrolysis in soybean (37). Soybean fermentation causes the hydrolysis of soybean protein into specific bioactive peptides (38). The latest research trend is to predict functional peptides in different types of vegetable proteins using emerging techniques, such as machine learning algorithms, and to predict the potential of several peptides (39, 40).

**Figure 1:**
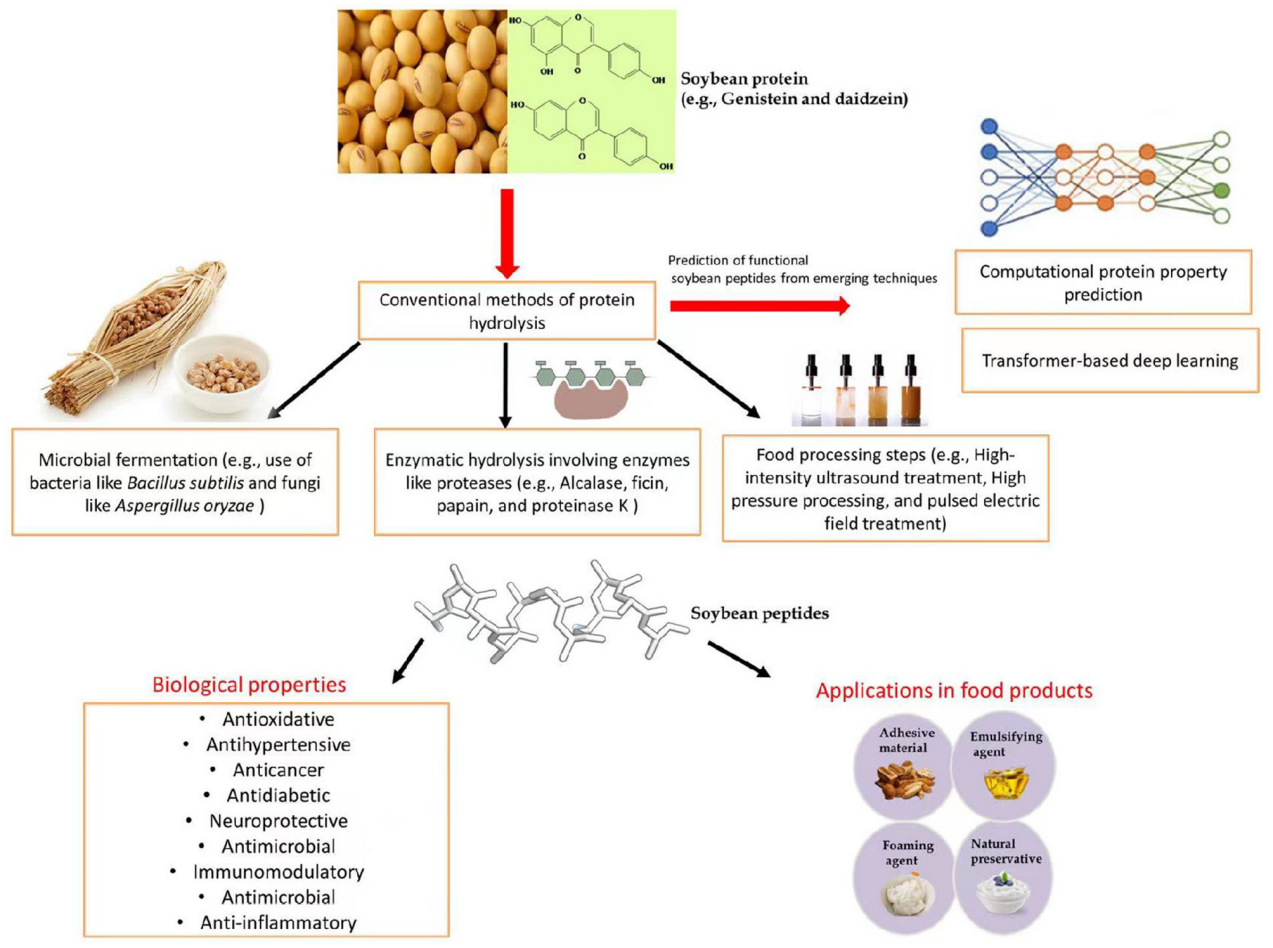
Functional applications of soybean-derived peptides.

The commercial use of soy protein in several food products is currently very popular, particularly in protein-based meat products for vegetarians (4). Figure 1 shows a general depiction of soybean-derived peptides in food products and their functional benefits.

However, the application of soybean peptides to food and feed still faces several challenges that hinder their effective use. The purpose of this review article is to provide recent updates on the functional and biological potential of soybean peptides and discuss conventional and emerging techniques to release these peptides from soybean. Finally, we discuss the practical applications of soybean peptides as functional ingredients in food and feed.

### Functional roles of soybean-derived peptides

Many soybean components have shown biological activity, including but not limited to proteins, peptides, saponins, and isoflavones (41). Soybean peptides have received special attention because of their huge diversity and associated biological potential. Several studies have revealed its anti-atherosclerotic, anticancer, antioxidative, anti-inflammatory, antiviral, antidiabetic, anti-inflammatory, and cardioprotective effects (Table 1).

**Table 1:** Health benefits of soybean-derived peptides.

**Soybean sources**	**Bioactive peptide sequence**	**Targeted model**	**Biological function**	**Outcomes**	**Reference**
Soybean glycinin	IAVPGEVA IAVPTGVA LPYP	HepG2 cells	Anti-hypocholesterolemic	Inhibit 3-hydroxymethylglutaryl coenzyme A (HMGCoA) reductase and stimulate the LDL receptor pathway which in turn reduces the cholesterol	(19)
Mature and young soybean proteins hydrolysates	FPFPRPPHQ, FMYL, MMLM, SFFFPFELPRE	Pancreatic lipase (PL) and cholesterol esterase (C-Ease) enzymes	Anti-lipidemic	Compared to flavorzyme and alcalase, bromelain showed higher levels of protein degradation, and its hydrolysis resulted in enhanced PL and C-Ease inhibition effectiveness. FPFPRPPHQ, FMYL, MMLM, and SFFFPFELPRE were effective inhibitors of the C-Ease enzyme. FPFPRPPHQ and SFFFPFELPRE had inhibitory potencies against both PL and C-Ease	(42)
Soybean	VHVV	Rats	Anti-hypertensive	Reduced p-PI3K, p-AKT, anti-apoptosis proteins (Bcl2 and Bcl-XL), SIRT1, and FOXO3 in rats. Activated cell survival, AMPKα1, Sirt1, PGC1α, and FoX3α proteins	(43, 44)
Soy protein hydrolysates	ILL LLL VHVV	HepG2 cells	Anti-obesity	Hydrolysates of soybean may reduce serum levels of triglycerides by decreasing fatty acid synthase (13.6) than control (17.0)	(6)
Soybean meal fermented by *Bacillus subtilis* E20	KHPHGRSYKTKLRILA LRFRAPAPVLRRIAKR HTSKALLDMLKRLGK	Shrimp	Anti-bacterial	Effective against microbes *Vibrio alginolyticus* and *V. parahaemolyticus*	(45, 46)
Soybean	PGTAVFK	*In vitro*	Anti-bacterial	At a concentration of 31 M, PGTAVFK also exhibits antibacterial action against *E. coli* and *S. aureus*	(47, 48)
Cheese peptidome	KFVPKQPNMIL	Glycoprotein receptor-binding domain (RBD) and main protease (3CLPro)	Anti-viral activity	Cheese peptidome could be used against the SARS-CoV-2 virus with binding energy values ranging from −8.45 to −26.8 kcal/mol and −15.22 to −22.85 kcal/mol to inhibit viruses' effect	(49)
Soy cheese fermented by *Lactobacillus delbrueckii* WS4	KFVPKQPNMIL	Human red blood cells	Anti-viral	Soy cheese is made with *Lb. delbrueckii* WS4 could be potential meal for SARS-CoV-2 and related viruses' prophylaxis	(49, 50)
Glycinin	IAVPTGVA LPYP	Caco-2 cells in the intestine of humans and HK-2 cells in the kidney	ACE enzyme inhibition	The activity of the ACE enzyme of renal and intestinal get decreased enzyme activity with desired IC_50_ values of 14.7 ± 0.28 and 5.0 ± 0.28 μM for Caco-2 cells, and 6.0 ± 0.35 and 6.8 ± 0.20 μM for HK-2 cells	(51, 52)
Soybean	PGTAVFK IKAFKEATKVVVVLWTA	*In vitro*	Anti-bacterial	Inhibitory concentration of 37.2 M, the peptide with long chain (IKAFKEATKVVVVLWTA) is more efficient in opposing *Listeria monocytogenes* and *Pseudomonas aeruginosa*	(53, 54)
Soybean protein concentrate	FPLLVLLGTVFLASVCVSLKVREDE NNPFYFR FFEITPEKN- PQLRDLDIFLSSVDINEGALLLPHFNSK	*In vitro*	Antioxidant and inhibitory assay	High molecular mass peptides demonstrated maximum antioxidant activity than low molecular mass peptides, however in case of inhibitory activities, low molecular peptides resulted better effects for α -glucosidase inhibition (IC50 = 0.94) and lipases (IC50 = 4.06) than high molecular peptides inhibition of α-glucosidase (IC50 = 3.4) and of pancreatic lipase (IC50 = 2.02)	(55)
Germinated soybean protein digest	VVNPDNNEN QEPQESQQ SDESTESETEQA	Human colon cancer cells, mouse macrophages RAW 264.7	Anticancer and anti-inflammatory effects	Fractions, 5–10 kDa peptides revealed maximum showed potency with IC50 = 11.70 mg/mL as compared to >10 kDa, which showed IC_50_ = 13.21 mg/mL values against cancer while 45% inhibition observed against inflammation	(56–58)
Soybean	SKWQHQQDSC RKQKQGVNLT PCEKHIMEKI QGRGDDDDDDD DD	Mice	Hypocholesterolemic	After 4 weeks of receiving lunasin with 0.125–0.5 μmol/kg·day dose mice had remarkably low PCSK9 and high amounts of LDLR levels in hepatic tissue, moreover, it decreased total-cholesterol (T-CHO), LDL-C in blood and up-regulated LDLR in HepG2 cells	(59)
β-conglycinin	YVVNPDNDEN	Mouse liver	Anti-obesity	Weight of adipose tissue reduced by increasing postprandial circulating FGF21	(60)
Soybean protein	EKPQQQSSRRGS	Mice	Immunomodulatory effect	Enhanced phagocytosis, retard excessive inflammatory response, and induced macrophages M1 polarization in the spleen of mice	(61)
Soy protein isolate (bromelain and thermolysin)	-	Human oral squamous carcinoma cell line, HSC-3	Anti-proliferative activity	After 72 h, both isolates showed inhibition against cancer cells which were 35.45–76.39%	(62)
Hydrolysates of soy protein Flavorzyme (F-SPIH)	VHVV	Hypertensive rats	Neuroprotective effect	A peptide that is bioactive VHVV had stimulated CREB-induced downstream proteins that might decrease the long-term memory loss mediated by hypertension and maintain the survival of neurons	(63, 64)
Black soy	–	*In vivo*	Blood pressure	Systolic blood pressure reduced clearly in peptide supplemented group (−9.69 ± 12.37 mm Hg) as compared to control (−2.91 ± 13.29 mm Hg)	(65)
Soy β-Conglycinin	YVVNPDNDEN YVVNPDNNEN	HepG2 Cells	Hypocholesterolemic	At 350 μM 1st peptide upregulates the mature SREBP2 protein level (134.0 ± 10.5%), enhanced the LDLR protein (152.0 ± 20.0%), and HMGCoA reductase protein (171 ± 29.9%), as compared to 2nd peptide where mature SREBP2 protein regulation was 158.0 ± 9.2%, the increase in LDL and HMGCoA reductase was 164.0 ± 17.9%, and 170 ± 50.0%, respectively	(66)
Glycinin	IAVPTGVA	Human intestinal Caco-2 cells and serum	Anti-diabetic	Stopped DPP-IV action *in situ*, with IC_50_ values 223.2 μM	(67, 68)
Glycinin and β-conglycinin,	–	Human intestinal Caco-2 cells	Hypocholesterolemic Anti-Diabetic	Peptides enhanced protein levels (51.5–63.0%, 55.2–85.8%) than control (0.5–1.0 mg/mL) moreover DPP-IV activity decreased (16.3–31.4% and 15.3–11.0%) than control (1.0 and 2.5 mg/mL)	(69)
Soybean protein isolate	FDPAL	*Caenorhabditis elegans*	Anti- oxidative	Caused a remarkably increase in resistance to oxidative stress, upregulated the specific expression of genes in *Caenorhabditis elegans*	(70)
Soybean glycinin	VAWWMY	Rats	Anti- hypocholesterolemic	Serum (0.03), liver (0.03), and intestine (0.2) detected low values of cholesterol	(71)
Soybean	QRPR	RAW 264.7	Anti-inflammation	Soybean peptide reduced the cytokines levels such as TNF-α and IL-6 that are responsible for inflammation	(72)
*Bacillus. licheniformis* KN1G mediated soybean	KFVPKQPNMIL	Human ACE2 cell receptor	Anti-viral	Preventative measures against SARS-CoV-2 infection	(16)
Soybean fermented by *Bacillus subtilis* (KN2B and KN2M).	ALPEEVIQHTFNLKSQ	*In silico*	Anti-viral	Effective against Severe Acute Respiratory Syndrome Coronavirus 2 (SARS-CoV-2).	(16)
Soybean induced peptide	QRPR	RAW264.7 cell model	Anti-inflammation	Autophagy was activated by QRPR in the inflammatory cell; however, autophagy was inhibited by reducing QRPR inhibitory effect	(73, 74)
R95, N98-4445A, S03-543CR	–	Human blood, breast, and prostate	Anti-proliferative activity	Showed up to 68.0% inhibition of cancer lines	(75)
Glycinin, β-conglycinin and lectin from soy milk	HSYNLRQSQVSELKYEGNWGPLV NPESQQGSPRV	*In vitro*	Anti-oxidative and anti-hypertensive effects	Peptides (10 kDa) resulted maximum antioxidant and DDPH values as 1,831 ± 20.29 TEAC μm and 50.74 ± 0.27%, respectively while ace inhibition was 75.97 ± 1.5%.	(76)
Soybean	VHVV	Rats	Antihypertensive	Antioxidant defense induced, stabilized mitochondrial homeostasis, decreased renal damage, induced free radicals in rats	(77)
Alcalase and neutrase	QNGEQE RGASADGPR YGGGGE	Macrophage RAW264.7 cells	Immunoregulatory effect	The cell viabilities decreased from 97.61 to 115.01% to 86.65%	(78)
Soybean protein	DGWFR ALSWLR DGWFRL PNGPVWR	Mice model	Sleep effects	Mice provided with 0.65 g kg^−1^ soybean-derived peptides showed 59.21% sleep duration on 3rd day of observation and increased melatonin levels (95.31%) while with 2.60 g kg^−1^ peptides doubled the serotonin in the brain and increased the awaking situation	(79)
Alcalase low molecular weight fraction (SPH-I, MW < 3 kDa)	VVFVDRL VIYVVDLR IYVVDLR IYVFVR	Human intestinal Caco-2 cells	Anti-oxidative effect	Desirable results showed in the case of ORAC (143 ± 2.1–171 ± 4.8 μM TE/μM), FRAP (54.7 ± 1.2–79.0 ± 0.6 mM Fe^2+^/μM), radical-scavenging activity (3.42 ± 0.2–4.24 ± 0.4 mMTE/μM) and DPPH (16.5 ± 0.5–20.3 ± 1.0 μM (TE)/μM). SPH-IC (85.9%) and SPH-ID (96.2%) restrained maximum H_2_O_2_-induced ROS generation	(80)
Lunasin, lectin	SKWQHQQDSCRKQKQGVNLTPCEK HIMEKIQGRGDDDDDDDDD	Cancer cells of the human breast	Anti-cancer effect	MDA-MB-231 cells were inhibited with these bioactive peptides (glycitein, Genistein, β-sitosterol, and genistin) while 142.67 ± 5.88 μM, 93.75 ± 5.15 μM, 196.28 ± 4.45 μM and 127.82 ± 4.70 μM and values were detected	(81)

Beyond its high nutritional value, soybean contains a large variety of useful substances, such as bioactive peptides. When soybean proteins break down, peptide pieces hidden inside their core assemblies are released. These peptide fragments exhibit a wide range of biological and functional properties. These peptides are produced from glycinin and beta-conglycinin (which includes three subunits, such as alpha, α′, and beta), the progenitors of the majority of isolated peptides (82). Fatty acid (FA) synthase (FAS) inhibition, triglyceride and cholesterol reduction, effectiveness against obesity and diabetes, and lipid metabolism enhancement are properties attributed to soybean peptides (83). Some studies have shown that soybean peptides have many functions, including hypocholesterolemia, outcomes against cancer, angiotensin-converting enzyme (ACE), hypertension, and regulation of the immune system, while additional uses and benefits are constantly being uncovered (84, 85). Immunomodulatory peptides belong to a complex class of biologically active peptides that contain substances with various mechanisms of action. Several studies have shown that soybean peptides exert immunoregulatory effects (Table 1). For instance, in the macrophage cell lines RAW 264.7 and THP-1 (86), lunasin displayed anti-inflammatory effects by reducing the generation of cyclooxygenase-2 (COX-2), E2 (PGE2), and nitric oxide (NO), as well as the expression of inducible NO synthase (iNOS). Additionally, interleukin-6 and interleukin-1 production was suppressed by lunasin, and its anti-inflammatory effects were linked to the repression of the NF-κB pathway. The ability of lunasin to inhibit integrins is likely responsible for its anti-inflammatory effects. The complete sequence is important for the anti-inflammatory benefits of lunasin fragments (21, 87). According to some studies, lunasin can reduce the production of tumor necrosis factor-alpha (TNF-α) which has anti-inflammatory effects. Lunasin demonstrates immunomodulatory activity against cancer by interacting with the cytokine's interleukin-2 (IL-2) and interleukin-12 (IL-12). This blend activates cells called natural killer (NK) cells to increase interferon-gamma (IFN-γ) production. Granzyme B (GZMB) and granulocyte-macrophage colony-stimulating factor (CSF2) expression were both elevated by a combination of lunasin/IL-2/IL-12, although transforming growth factor beta receptor 2 (TGFBR2) and transforming growth factor beta 1 (TGFB1) expression were decreased (88). As a result, the ability of lunasin to modulate gene expression is associated with its immunomodulatory properties. The combination of lunasin and IL-12 significantly increased H_3_ acetylation at the interferon gamma (IFNG) locus and decreased it at the TGFB1 locus, indicating an epigenetic mechanism (18). A peptide without either the RGD motif or the aspartic acid (D)-tail, however, had an impact comparable to that of full-length lunasin, indicating that they were not linked to a synergistic augmentation of IFN production. Therefore, the N-terminus and/or central regions of lunasin are linked to the immunomodulatory function of the compound. The reduction in cholesterol levels is attributed to lactostatin, which is β-lactoglobulin (IIAEK), which regulates these levels by controlling the channels of the calcium-associated signaling pathway of MAPK. Cholesterol 7α-hydroxylase (CYP7A1) is the limiting enzyme involved in the degradation of cholesterol and cells of HepG2, mediating transactivation, which is induced by lactostatin. The activity of this enzyme is controlled by calcium channels and the extracellular signal-regulated kinase (ERK) pathway (60). Various peptides have been shown to play important roles in lowering cholesterol and lipid content (Table 1). Glycinin hydrolysis with trypsin and pepsin revealed two peptides that were converted into IAVPTGVA (Soy1) and LPYP peptides, which were shown to be absorbed by Caco-2 cells (89). They either act as hypocholesterolemic or hypoglycemic agents and can inhibit HMGCoA reductase and stimulate the LDL receptor pathway, which, in turn, reduces cholesterol. Additionally, there was an increase in the intensity of phosphorylation on Ser 872 of HMGCoA reductase, which is the operative form of HMGCoA reductase, through the stimulation of the adenosine monophosphate-activated protein kinase (AMPK) pathway. Stimulation of AMPK and Akt/protein kinase B pathways is linked to the ability to regulate glucose metabolism and uptake (89). Inhibition of glycogen synthase (GS) and glycogen synthase kinase-3β (GSK3) is caused by the activation of Akt (phosphorylation at Ser 473), which further controls the activity of GS by modifying glycogen formation in hepatic cells. In addition, the upregulation effect of hepatocytes on extracellular free glucose is also determined by the expression level of glucose transporter type 1 (GLUT1) and glucose transporter 4 (GLUT4).

Different protein hydrolysates can be produced by enzymatic hydrolysis of mature and young soybean proteins using bromelain, alcalase, and flavourzyme at various hydrolysis reaction times (42). The antilipidemic qualities of the hydrolysates were assessed based on their capacity to inhibit pancreatic lipase (PL) and cholesterol esterase (C-Ease) enzymes. Using a liquid chromatography-mass spectrometry quadrupole time-of-flight system (LC-MS QTOF) and the prediction of molecular interaction mechanisms, additional analysis was performed on the chosen hydrolysates for peptide identification. The purpose of this study was to determine if the PL and C-Ease enzymes can be inhibited by mature soybean protein hydrolysates (MSPHs) and young soybean enzymatic protein hydrolysates (YSPHs). Compared to flavourzyme and alcalase, bromelain showed higher levels of protein degradation, and its hydrolysis resulted in enhanced PL and C-Ease inhibition effectiveness. Using LC-MS QTOF and molecular binding investigations, six PHs with strong antilipidemic effects were selected for sequencing. It was predicted that the peptides would have strong inhibitory effects on PL. Additionally, it was projected that these compounds would be effective against C-Ease. When tested against PL and C-Ease, they showed strong inhibitory potential. Soybean-derived protein hydrolysates from mature and young beans have been identified as a possible component in the treatment of hypercholesterolemia. Based on these findings, hydrolyzing MSPI and YSPI with alcalase, bromelain, and flavorzyme encouraged the production of powerful PHs rich in bioactive peptides that may inhibit PL and C-Ease. In contrast to YPHs, MSPHs produced by enzymes showed stronger PL inhibition; however, YSPHs produced by enzymes showed higher C-Ease inhibitory capabilities. Bioactive peptides produced from MSPHs and YSPHs were capable of suppressing PL and C-Ease, according to *in silico* molecular binding investigations. Overall, the peptides generated from the MSPHs and YSPHs showed strong inhibitory effects against PL. In addition, it was predicted that the peptides FPFPRPPHQ, FMYL, MMLM, and SFFFPFELPRE from MSPHs and YSPHs would be effective inhibitors of C-Ease. Furthermore, it was projected that the peptides FPFPRPPHQ and SFFFPFELPRE, which were generated from MSPHs and YSPHs, respectively, would have inhibitory potencies against both PL and C-Ease, indicating that these peptides would have dual functions against antilipidemic enzymes. The curative benefits of proteins obtained from soybean, which are mature and young, make it an intriguing candidate for use as an efficient component in the creation of anti-lipidemic functional products (42).

Superoxide anions (O_2−_), hydrogen peroxide (H_2_O_2_), and hydroxyl radicals are examples of reactive oxygen species (ROS) that are essential for everyday bodily functions (OH). However, when ROS accumulates beyond the capacity of the cellular antioxidant defense system, metabolic disorders such as cardiovascular disease, Alzheimer's disease, type 2 diabetes (T2D), and certain types of cancer may develop (90, 91). Additionally, ROS are produced chemically or enzymatically in food systems, and their interactions with dietary ingredients result in unfavorable tastes and carcinogens. Therefore, soy protein hydrolysates and constitutive peptides have been employed to prevent food systems from degrading due to ROS and to shield the human body from harmful effects. The fraction with the lowest molecular weight (SPH-I, MW3 kDa) demonstrated the strongest radical scavenging ability and reducing power as well as the best potency in controlling H_2_O_2_-induced oxidative stress in Caco-2 cells (92). It would be interesting to isolate bioactive peptides with cytoprotective and antioxidant properties. These peptides also greatly reduced lipid peroxidation and intracellular ROS formation, which protected Caco-2 cells from H_2_O_2_-induced oxidative damage (*p* < 0.05). Furthermore, SPH-IC and SPH-ID significantly increased the ROS-mediated response to inflammation by preventing interleukin-8 release (*p* > 0.05) (80). The amino acid makeup of lunasin has been linked to its antioxidant action. In a study, lunasin was shown to decrease lipid peroxidation, shown 2, 2′-azino-bis (3-ethylbenzothiazoline-6-sulfonic acid) radical scavenging activity (ABTS•+), and prevent the production of ROS caused by lipopolysaccharide. After scavenging peroxyl and superoxide radicals, chelating ferrous ions, and reducing intracellular ROS levels, the antioxidant activity of lunasin was further proven (90, 93). According to Fernández-Tomé et al. (94), under oxidative stress, lunasin inhibits an increase in the activity of glutathione peroxidase and catalase, lowers intracellular ROS and protein carbonyl levels, and increases cytosolic glutathione levels. In some experiments, the inhibition of dipeptidyl peptidase-IV (DPP-IV) activity by Soy1 and LPYP is a favorable outcome for the prevention of diabetes (95). Using BIOPEP, an initial examination of their structures indicated that angiotensin-converting enzymes might be potent inhibitors. Consequently, a bottom-side-up approach was established to clarify hypotensive activity *in vitro*. Similarly, in another study using molecular modeling, their capacity to act as inhibitors was competitive with this enzyme (51). Production of low insulin or resistance to insulin causes dysregulation of the glucose balance which results in T2D. To manage T2D, a considerable focus is on natural remedies, especially food elements. DPP-IV, alpha-amylase, and alpha-glucosidase are essential for controlling blood glucose levels. It is believed that the suppression of these enzymes by bioactive peptides could be the most efficient method for managing T2D. The peptides obtained from food are being focused on as novel inhibitors because DPP-IV, which is present in the cell membrane and blood, is responsible for the inhibition of incretin hormones, such as stomach inhibitory polypeptide and glucagon-like peptide-1 (72). For the efficient control of T2D and other metabolic disorders, the inhibitory peptide DPP-IV (IAVPTGVA) obtained from soybean may be utilized.

### Emerging tools and techniques for the exploration of functional peptides in soybean

Small protein fragments known as soy peptides are produced through enzymatic hydrolysis *in vitro*, fermentation (such as fermentation caused by bacteria containing lactic acid), processing of food such as modification of pH, heat treatment, isolation of protein, processing through ultra-high-pressure (96, 97), and GI digestion (specific and non-specific proteases from the pancreas, small intestine, and stomach, including pepsin, trypsin, and chymotrypsin). Table 2 shows the different methods used to produce soybean peptides. Soy protein also affects peptide composition through enzymatic hydrolysis or bacteria-mediated fermentation (6). Diverse functional features of soy peptides with various compositions have also been noted when producing tofu in terms of quality, yield, and texture (109). The digestion of food in the digestive tract can also produce peptides. Proteins are hydrolyzed by digestive enzymes, resulting in peptides of various lengths and free amino acids. Pepsin acts at the stomach level in *in vitro* systems, randomly hydrolyzing peptide bonds to create relatively large peptides and a mixture of acids from the pancreas and pancreatins. Trypsin, chymotrypsin, elastase, and carboxypeptidases are the only peptidases that constitute pancreatins. Except for trypsin, all enzymes hydrolyze peptide bonds, resulting in peptides with various amino acid sequences (110).

**Table 2:** Various methods for soybean-based peptide generation.

**Methods**	**Species for hydrolysis**	**Findings**	**Peptide structure**	**Type of the trial**	**References**
Microbial fermentation	*Lactobacillus delbrueckii* WS4	The binding energy was −8.45 to −26.8 kcal/mol and −15.22 to −22.85 kcal/mol	KFVPKQPNMIL	*In vitro*	(49, 98)
Enzymatic hydrolysis	Corolase PP	The degree of hydrolysis enhanced to 18.9% ± 1.9 after 10 h, Significant increase in antioxidant and ACE inhibitory effects	FEITPEKNPQLRDLDIFLSI INAENNQRNFLAGSQDNVISQIPSQV FAIGINAENNQRNFLAGSQDNVISQIPSQV	*In vitro*	(99)
Enzymatic hydrolysis	Corolase PP	Showed protective effect against hypertension and potent antioxidant activity	IRHFNEGDVLVIPPGVPY, IRHFNEGDVLVIPPGVPYW, IYNFREGDLIAVPTG, VSIIDTNSLENQLDQMPRR, YRAELSEQDIFVIPAG	*In silico*	(100)
Enzymatic hydrolysis and lactic acid fermentation	Prozyme and *Lactobacillus rhamnosus* EBD1	ACE inhibitory activity enhanced (60.8 ± 2.0%−88.24 ± 3.2%), moreover captopril showed an inhibitory effect (94.20 ± 5.4%)	PPNNNPASPSFSSSS GPKALPII IIRCTGC	*In vitro*	(101)
Microbial fermentation	Proteases and *Enterococcus faecium*	The ACE inhibitory ability increased from 15 ± 3% to 40 ± 2% by treating soybean with Prozyme 2000p while subsequent fermentation of the hydrolyzed samples by *E. faecium* further increased (80 ± 5%) and improved the antihypertensive peptides, phenolic compounds, gamma-aminobutyric acid, and antioxidants levels	ENPFNL EDEVSFSP RSPFNL SRPFNL	*In vitro*	(102)
Enzymatic hydrolysis and ultrasound pretreatment	Trypsin	Fraction < 5 kDa demonstrated the maximum inhibitory effect (0.27 mg/mL) and >5 kDa fraction resulted the least inhibitory activity (3.31 mg/mL)	Gly-Ser-Arg and Glu-Ala-Lys	*In vitro*	(103)
Pulsed electric field	5 kV/cm, 2,400 Hz, and 2 h	DPPH remarkable enhanced (*P* < 0.05) to 94.35 ± 0.03%, while zeta potential decreased (0.59 ± 0.03 mV)	SHCMN	*In vitro*	(104)
Enzymatic hydrolysis	Corolase L10 (Cor), Promod 144MG (Prom) and Protamex (Prot)	IC50 amounts were 0.73 ± 0.11 to 3.54 ± 0.24 mg	LPQNIPPL	*In vitro*	(105)
Enzymatic hydrolysis	GI enzymes and Alcalase	Effectiveness in anti-proliferation against human blood, prostate cells, and breast cancer	KWKLFKKIPKFLHLAKKF	*In silico*	(75)
Ultrasound-assisted liquid-state fermentation	*B.subtilis*	Increase in ACE inhibitory, peptides content and biomass were 26.4, 36.2, and 55.0%, respectively	Gly-Gly-Tyr-Arg	*In vitro*	(106)
Microbial fermentation	*B.licheniformis* KN1G, *Bacillus amyloliquefaciens* KN2G and *B.subtilis* (KN2B and KN2M)	Effective inhibition of SARS-CoV-2 S1 receptor binding domain and the modulation of Toll-Like receptor 4	ALPEEVIQHTFNLKSQ	*In silico*	(16)
Microbial fermentation	*Lactobacillus plantarum* strain C2	Maximum peptide showed in 10 kDa fractions and resulted in highest ace inhibition (73.35 ± 1.5), ABTS (1,831 ± 20.29 TEAC μm) and DPPH (50.74 ± 0.27%) effects. Total of 51 peptides discovered	ESYFVDAQPKKKEEGNK SLKVREDENNPFYFRSSNS HSYNLRQSQVSELKYEG NWGPLVNPESQQGSPRV	*In vitro*	(76)
Microbial	*Lacticaseibacillus casei* (NK9) and Lacticaseibacillus *fermentum* (M2)	Higher ACE-inhibitory (48.44%) and proteolytic activity (0.67 OD) is seen in soy milk fermented with M2 than NK9 (proteolytic activity: 0.48 OD and ACE-inhibitory activity: 41.33%)	SGLGRGWIDGDIGHGK SMEDMM VPVVLGSKNEVDYIK GYHYVGTLSGHTK VREDGV YCEIVPFQK TPPASWSKLGYK	*In vitro*	(107)
Enzymatic hydrolysis	Alcalase	Protect Caco-2 cells from H_2_O_2_-induced oxidative damage, and reduced ROS-mediated inflammatory reactions by preventing the interleukin-8 release	VVFVDRL VIYVVDLR IYVVDLR IYVFVR	*In silico*	(80)
Enzymatic hydrolysis	Pepsin, chymotrypsin, and trypsin	Showed no cytotoxic effects on HEK293 with IC_50_ value of 3.95 ± 0.11 mM	DMG	*In silico*	(108)

Chemical-based techniques and fermentation with microbes are the conventional methods used for hydrolyzing soybean peptides. There are a few examples of the chemical-based approaches mentioned above (Table 2). Advanced fermentation methods (fungus, yeast, or germ) and enzymatic treatments are two examples of processing technology (111). In addition to physical techniques, recent studies have highlighted the significance of enzymatic hydrolysis. Peptides, considered biowaste, are obtained from sources of protein by hydrolysis through enzymes and have been used as an assuring method. It has been reported that a meal of soybean contains two soybean lines that are high in oleic acid and one high-protein line when utilized from peptides are bioactive and are effective against cancer cells at multiple sites. GI enzymes and alcalase are used to resist GI to obtain fractions of soy peptides (75).

A viscozyme multi-enzyme complex was employed by de Figueiredo et al. (112) to pretreat okara (soybean waste product), which enhanced the conventional alkaline preparation procedure and increased the protein extraction rate. The major goal of this study was to suggest three techniques for producing low-molecular-weight peptides from okara. High-pressure homogenization and mixed enzyme hydrolysis were followed by alkaline protease hydrolysis and the alkali-dissolved acid precipitation method for alkaline protease hydrolysis and protein extraction, respectively. The findings of this study were an increase in the added value of okara and the production of biologically active peptides from soybean waste. In another study, a method for producing low-molecular-weight peptides (HPH-VAP) from okara was proposed using high-pressure homogenization-assisted double enzymes. To compare the effects of the various procedures, the rates of protein extraction, peptide structure, antioxidant capacity, and immunological characteristics were evaluated. The results demonstrated that the protein extraction rate of this method increased by 69 and 51%, compared to earlier methods. The results showed that it increased NO levels, cytokine production, and cell phagocytic capabilities (IL-6, IFN–γ, and TNF-α) (113).

Soybean fermentation breaks down soybean proteins into small peptides. The breakdown of proteins during soybean fermentation may be accelerated by enzyme hydrolysis, thereby improving peptide output. In a previous study, soy meal that had been fermented with Douchian *B. subtilis* was hydrolyzed using the enzyme thermolysin. Subsequently, four fractions of the extracted water were separated using ultrafiltration membranes. Following the evaluation of vasorelaxation capabilities, the most effective fraction was isolated and purified to obtain four peptides. Three peptides led to dose-dependent vasorelaxation (0.01–4.10 M) in the thoracic aorta ring of Sprague–Dawley rats (relaxation activities were all endothelium-independent), whereas one peptide led to vasoconstriction. Additionally, it was shown that the activities of ACE inhibition and vasorelaxation had a different causal relationship (114, 115). The goal of another study was to purify and identify antioxidant peptides from alcalase-hydrolyzed soybean (*G. max L*.) hydrolysate's low-molecular-weight fraction (SPH-I, MW3 kDa) and to further assess the peptides' ability to protect human intestinal Caco-2 cells from oxidative stress. The four main peptides were purified using reversed-phase HPLC and gel filtration chromatography, and their sequences were determined using nano-LC-ESI-MS/MS as follows: IYVVDLR (877 Da), VIYVVDLR (976 Da, SPH-IB), IYVFVR (795 Da, SPH-ID), and VVFVDRL (847 Da, SPH-IA and SPH-IC). The antioxidant peptides were created and showed promising antioxidant action against DPPH radicals [16.5 ± 0.5–20.3 ± 1.0 M Trolox equivalent (TE)/M], ABTS•+ radicals (3.42 ± 0.2–4.24 ± 0.4 mM TE/M), ORAC (143 ± 2.1–171 ± 4.8 M TE/M), and FRAP (54.7–79.0). Additionally, the peptides dramatically reduced lipid peroxidation and intracellular ROS formation, protecting Caco-2 cells from H_2_O_2_-induced oxidative damage. Additionally, SPH-IC and SPH-ID significantly increased total reduced glutathione synthesis, improved catalase, and glutathione reductase activities, and reduced ROS-mediated inflammatory reactions by preventing interleukin-8 release (80). In another experiment, antioxidant and antibacterial peptides were constantly produced from soybean milk in a membrane bioreactor. It was determined that using a static turbulence promoter in the membrane separation process resulted in a greater permeate flow and less energy being consumed in the filtering process. Using the static turbulence promoter throughout the membrane separation process at a constant operating transmembrane pressure of 3 bar and retentate flow rate of 100 L/h increased the permeate flux. The energy consumption was also reduced by the filtering process employed by a static turbulence booster. Both membrane permeate and enzyme-hydrolyzed milk have antioxidant and antibacterial activities against *B. cereus* (116). ACE-inhibitory tripeptides from soybean protein were determined using *in vitro* and *in silico* analyses. RP-HPLC was used to confirm the *in vitro* activity of the hypothetical ACE-inhibitory tripeptides. DMG was selected as a powerful ACE-inhibiting peptide. In a cell experiment, DMG was shown to have no cytotoxic effects on HEK293 cells. Furthermore, molecular docking results showed that DMG made good contact with the ACE active sites (His513, Ala354, Gln281, His353, Tyr520Glu384, and Lys511). Additionally, DMG showed significant anti-ACE activity with an IC_50_ value of 3.95 0.11 mM (108). Soybean protein hydrolysates were produced using two proteolytic enzymes (Alcalase and Protamex), and their functional and antioxidant properties, as well as the degree of hydrolysis (DH), were evaluated. The highest DH value was 20%, with a yield of 19.77% and protein content of 51.64%. More than 41% of the total amino acid composition of each protein was present in the hydrolysate. The protein hydrolysates from Protamex had excellent solubility, emulsifying activity, and foaming capacity at pH 2.0, with values of 83.83%, 95.03 m^2^/g, and 93.84%, respectively. The water-holding capacity of Alcalase was 4.52 g/g, whereas the oil-holding capacity of Protamex was 4.91 g/g. When samples were tested using DPPH (2, 2-diphenyl-1-picrylhydrazyl), Protamex showed the highest antioxidant activity (62.07%) and the lowest reducing power (0.27). Alcalase showed 70.21% ABTS activity. These findings show that soybean protein hydrolysates have substantial potential to improve the nutritional value, antioxidant activity, and functional properties of soybeans (117).

Different fermenting cultures have been used to hydrolyze soybean proteins (for example, *Bacillus* species). Soybean proteins are used extensively as functional and nutritional food ingredients. The effects of three factors [temperature, pH, and enzyme/substrate (E/S) ratio] on the production of soy protein isolate (SPI) hydrolysates using a microbial protease were estimated using a 23-central composite design. They evaluated the antioxidant activity, foaming, emulsifying potential, and soluble peptide content of the hydrolysates. The optimal conditions for producing soluble peptides were pH 6.5–9.5, 30–35°C, and E/S ratios of 1,650–6,300 UgL. The SPI hydrolysates produced showed a greater ability to chase the ABTS radical at pH 8.0–9.5, 30–45°C, and E/S ratios of 4,000–8,000 U g1p. These factors had no noticeable effect on the capacity of hydrolysates to scavenge the 2, 2-diphenyl-1-picrylhydrazyl (DPPH) radical in the examined range. SPI hydrolysates also demonstrate the capacity to chelate iron and their ability to decrease. The hydrolysis temperature was important for the capacity of the hydrolysates to chelate Fe2+. The findings of this research suggest that precise hydrolysis conditions should be carefully selected to produce SPI hydrolysates with appropriate properties (118). While processing soybeans with subsequent fermentation and proteases, the release of bioactive substances that can be firmly bound to the food matrix increases. This study compared the capacity of raw soybean, Prozyme 2000p-hydrolyzed soybean (PSB), and *Enterococcus faecium* EBD1-fermented PSB to inhibit angiotensin-converting enzyme to create an antihypertensive functional food from soybean. When soybean was treated with Prozyme 2000p, the ACE inhibitory capacity increased from 15 to 40%. The hydrolyzed samples were then fermented, which further boosted their inhibitory ability to 80.5%. A discovery-based metabolomic method utilizing UHPLC Q-TOF MS/MS of the fermented sample demonstrated enhanced levels of gamma-aminobutyric acid, antihypertensive peptides, phenolic compounds, and antioxidants owing to *E. faecium* fermentation. Fermentation increased the concentrations of essential amino acids compared to the raw and enzyme-treated samples. High concentrations of putative antihypertensive chemicals in the fermented samples suggested that fermenting soybean treated with Prozyme with *E. faecium* EBD1 might be a good method for producing functional foods with these hypertensive qualities (102).

This study evaluated the impact of fermentation on the nutritional value of food and food-grade soybeans. Both were fermented for 48 h by *Aspergillus oryzae* GB-107 in a bed-packed solid fermenter. After fermentation, nutritional and trypsin inhibitor levels were compared to those of soybean meal and raw soybeans. Compared to raw soybeans and soybean meals, fermented soybeans and soybean meals contained 10% higher crude protein (*P* < 0.05). The essential amino acid profile remained unchanged during the fermentation. Most trypsin inhibitors were removed by fermentation of both soybeans and soybean meal (*P* < 0.05). Although fermentation significantly reduced the number of large peptides (60 kDa) compared to raw soybeans, it significantly increased the number of small peptides (20 kDa) (*P* < 0.05). Fermented soybean meal contained more small peptides (20 kDa) than soybean meal (*P* < 0.01); however, soybean meal contained 22.1% larger peptides (60 kDa) than fermented soybean meal. In general, fermentation reduces the peptide size in soybeans and soybean meals, eliminates trypsin inhibitors, and increases protein content (108). The purpose of this study was to assess the bioactivities, such as galactosidase and glucosidase activities, and the growth behavior of *Lactobacillus* cultures in the soymilk medium. Among the 10 *Lactobacillus* cultures in the soymilk medium used in this study, *L. casei* (NK9) and *L. fermentum* (M2) were chosen because of their improved growth patterns and higher levels of glucosidase and galactosidase activities during fermentation. Additionally, soymilk fermented with M2 had stronger ACE-inhibitory (48.44%) proteolytic activity (0.67 OD) than NK9 (proteolytic activity: 0.48 OD and ACE-inhibitory activity:41.33%) (9). Using specific *Lactobacillus* cultures during the fermentation of soy milk, peptides were produced that were recognized by MALDI-TOF spectrometry as having effective ACE-inhibitory activity. Using the BIOPEP database, the identified ACE-inhibitory peptide arrangements from fermented soymilk were characterized (107).

IAVPTGVA (Soy1) and LPYP, two soybean peptides with multiple behavioral functions, have shown hypoglycemic and hypocholesterolemic effects *in vitro*. According to a preliminary structural screening conducted using BIOPEP, they may be significant ACE inhibitors. As a result, a bottom-up method was established to explain the *in vitro* hypotensive activity. With IC50 values of 14.7 ± 0.28 and 5.0 ± 0.28 M (Caco-2 cells) and 6.0 ± 0.35 and 6.8 ± 0.20 M (HK-2 cells), correspondingly, LPYP and Soy1 decreased the renal and intestine ACE enzyme activity. In addition, molecular modeling studies have suggested that they have the potential to function as competitive inhibitors of this enzyme. To improve stability and hypotensive qualities, a viable method for the non-toxic regulation of their release from a nanomaterial was devised by encapsulation into a RADA16-assembling peptide (51). AHTPs have been perceived in various organisms, and their anti-hypertensive activity in the laboratory for the identification of peptides is time- and resource-consuming. Before verification through experiments, computational techniques that comprise the stout learning of machines can recognize capable AHTPs. The research proposed Ensemble-AHTPpred, a collective learning of a machine-containing algorithm comprising maximum gradient boosting (XGB), a support vector machine (SVM), and a random forest (RF), to enhance the robustness of the final predictive model and incorporate various heterogeneous algorithms. To analyze or explain the characteristics of the predicted peptide, computed features such as transitions, n-grams, various physicochemical properties, secondary structure-related information, and amino acid composition (AACs) were used. Above 90%, was achieved on the liberated inspection data using the Ensemble-AHTPpred tool. Furthermore, based on the latest studies, the method was practical for innovative empirically authorized AHTPs that were not overlaid with the test and datasets that are based on training, and these AHTPs might specifically be predicted by the tool (119).

### Functional applications of soybean peptides in food and feed

Soybean-derived peptides have gained great popularity as one of the most economical and easily accessible peptides, with a plethora of functional applications in the food, feed, and pharmaceutical industries. Thermal and gastrointestinal stability (including pH) are crucial parameters for the practical application of soybean peptides in food and feed (120). A study reported that the interfacial and emulsifying characteristics of soy peptides varied based on the degree of hydrolysis. A peptide with the lowest degree of hydrolysis was found to be an excellent functional agent for the emulsification of not only silicon oil and liquid paraffin but also soybean oil (121). Novel nanoparticles derived from soy peptides have shown great potential as oil–water emulsion stabilizers and effective food-grade emulsifiers, with the potential to replace surfactants and polymers (122). These studies indicate that soy peptides had previously unappreciated emulsifying activity and great interfacial properties, which make them useful for the food processing industry. Soy peptides have exceptional assimilating properties, and there is a need to determine whether the foaming properties of egg white powder can be enhanced by using soy peptides as foaming agents. There is some evidence that 9–12% of soy peptides at pH 7 improve the foaming properties of egg white powder by conferring it a more flexible secondary structure, uniform size, more surface hydrophobicity, and foam elasticity (123).

The effect of soybean-derived peptides on the quality attributes (physicochemical, sensory, and microbiological) of yogurt was evaluated under storage (3 weeks) conditions. The enzymatic hydrolysis of soy whey protein was performed using trypsin at 45°C for 4 h. Various concentrations of soy peptides (6.5, 13, and 17 mg/mL) were incorporated into yogurt. Increasing the peptide content enhanced antioxidant activity; however, viscosity and syneresis were reduced. During 3 weeks of storage, acidity (from 1.04 to 1.14%), syneresis (from 15.23 to 19.84%), and viscosity (from 5.31 to 8.04) of yogurt increased while pH (from 4.54 to 4.37) and antioxidant activity (from 12.55 to 9.32) decreased. The incorporation of 13 mg/mL of peptide showed a maximum decline in *Escherichia coli* (0.25 CFU/mL) and *Staphylococcus aureus* (0.79 CFU/mL) levels. Therefore, we conclude that soy whey-derived peptides can be used as a natural preservative of yogurt (124).

A previous study determined the thermal and pH stability of antioxidative and ACE-inhibitory peptides derived from soybean peptides after fermentation with *L. plantarum* strain C2. It was concluded that the peptides were thermally stable over a wide temperature range (25–121°C) and pH range (4, 6–8, 13, 14). These stability features indicate the potential thermal resistance of soy peptides in food processing and gastrointestinal digestion at low pH (76). Soy-derived bioactive peptides or soy protein isolates are relatively more economical, even when compared to peptides derived from different dairy proteins (125). They have been reported to promote the growth of some probiotic bacteria, making them a potential prebiotic in food and feed. For instance, *Lacticaseibacillus rhamnosus* has been shown to utilize both hydrophobic (with 3–5 amino acid residues) and hydrophilic peptides (with >5 amino acid residues) (126). Similarly, soy peptides and proteins have been reported to promote growth and confer competitive advantages to *Lactobacillus rhamnosus* over *Escherichia coli* (127, 128). Furthermore, the antimicrobial potential of soybean peptides (53, 128) makes them a great additive to enhance the shelf-life of food and various animal feeds. Kefir is fermented milk that has been consumed for thousands of years. It originated in parts of Eastern Europe and the lush regions of the Caucasus Mountains. Isoflavone biotransformation and flavor production during soymilk kefir fermentation were found to be cultivar- and culture-specific (129–131).

## Conclusion

The increasing trend in scientists exploring soybean, their proteins, the synthesis of bioactive peptides, and the discovery of new health benefits associated with soybean will surely help improve human health. The consumption of soy products is promising for reducing various chronic diseases, such as cancer and diabetes. These health benefits are associated with the raw soy protein or derived soy bioactive peptides obtained by processing. Bioactive peptides are primarily generated by enzymes, fermentation, and gastrointestinal digestion. Soy peptides' bioregulatory mechanisms, structural configuration, and mechanism identification are in the developing stage; therefore, research should be conducted on a large scale to identify all of these aspects. This research will lead to the identification of new bioactive peptides and new roles in health, and hence, will improve public health.

## Author contributions

YZ drafted the manuscript. GC collected the data and results. CW and JD modified the manuscript. All authors contributed to the article and approved the submitted version.
